# Individual Variations in Vergence and Accommodation Responses Following Virtual Reality Gameplay

**DOI:** 10.3390/vision8040069

**Published:** 2024-12-20

**Authors:** Alona Sumarokova, Reinis Alksnis, Dagni Rappo, Karola Panke, Gunta Krumina, Tatjana Pladere

**Affiliations:** 1 Department of Optometry and Vision Science, Faculty of Science and Technology, University of Latvia, LV-1004 Riga, Latvia; alona.sumarokova@lu.lv (A.S.); karola.panke@lu.lv (K.P.); gunta.krumina@lu.lv (G.K.); tatjana.pladere@lu.lv (T.P.); 2 Laboratory of Statistical Research and Data Analysis, Faculty of Science and Technology, University of Latvia, LV-1004 Riga, Latvia; reinis.alksnis@lu.lv; 3 Medical Technology Education Centre, Tallinn Health Care College, 13418 Tallinn, Estonia

**Keywords:** virtual reality, visual functions, binocular and accommodative disorders, refraction shift, accommodation lag, accommodative microfluctuations

## Abstract

Virtual reality (VR) can challenge the visual system, leading to temporary oculomotor changes, though the degree of change varies among individuals. While the vergence and accommodation system plays a crucial role in VR perception, it remains unclear whether individuals whose visual functions fall outside clinical norms experience larger changes. Thus, our study aimed to investigate whether changes in vergence and accommodation responses following VR gameplay differ between individuals with and without non-strabismic binocular and accommodative disorders. To assess this, both subjective and objective measurements were conducted before and after 20 min of playing Beat Saber. Results revealed significant alterations across both subjective measurements—near point of convergence and near point of accommodation—and objective measurements, including eye refraction, accommodation lag, and accommodative microfluctuations at far. Moreover, individuals with non-strabismic binocular and accommodative disorders exhibited larger accommodative microfluctuations at far compared to the control group. Overall, these findings indicate that considering individual differences in vergence and accommodation responses is important when evaluating the impact of VR on the visual system and can be helpful in the design and use of VR systems, particularly for individuals with binocular and accommodative disorders.

## 1. Introduction

The growing use of virtual reality (VR) technology has raised concerns in eye care regarding its potential impact on vergence and accommodation. Conventional VR head-mounted displays employ a stereoscopic approach to visualize information, simulating three-dimensional images. In such systems, images can appear closer or farther away from the focal plane depending on the horizontal displacement of images for both eyes. Consequently, to perceive a fused and in-focus image, the eyes must converge at the image location while accommodating at the focal plane. Considering vergence and accommodation are neurologically coupled processes [1], this viewing condition can lead to a sensory conflict, visual stress, and altered functionality of the visual system [2,3].

When analyzing general group behavior, temporary oculomotor changes, such as reductions in near point of convergence (NPC) and near point of accommodation (NPA), have been reported following exposure ranging from 10 to 120 min using different VR devices and applications [4,5,6,7,8,9]. Nevertheless, a closer examination of individual data available in some studies reveals considerable variations in vergence and accommodation responses. Specifically, both decreases and increases have been observed in NPC, NPA [5], and accommodative amplitude [10] following VR exposure, while in some individuals, these parameters remained unchanged. However, these variations are typically masked in analyses solely focusing on general group behavior [11], leaving the underlying reasons understudied.

The use of headsets with near-eye displays can challenge the visual system, with the outcome potentially depending on the individual range of optical misalignments and sensory conflicts that the visual system is able to overcome [12]. Thus, differences in baseline vergence and accommodation measurements might be related to variations in oculomotor changes following VR exposure.

Non-strabismic binocular and accommodative disorders affect at least one-third of young people [13,14,15,16,17,18,19], some of which remain undiagnosed [14,20,21]. Given the frequency of these disorders, it is important to elucidate how vergence and accommodation responses may be altered in individuals with these disorders following VR exposure compared to those without such disorders.

As VR often imposes greater demands on the visual system than other screen-based activities, particularly in dynamic scenarios requiring frequent shifts in vergence angles [2,6], this study investigated whether changes in vergence and accommodation responses following VR gameplay differ between individuals with and without non-strabismic binocular and accommodative disorders, using both subjective and objective measurements. A better understanding of individual variations in vergence and accommodation could contribute to developing more inclusive VR experiences, ensuring accessibility for a diverse range of users in the future.

## 2. Materials and Methods

### 2.1. Participants

A total of 87 individuals volunteered to participate in this study, of whom 62 (49 women and 13 men, mean age ± SD: 22 ± 4 years) met the inclusion criteria. The inclusion criteria were as follows: binocular and monocular visual acuity at far and near of at least 1.0 decimal units (measured with the Snellen chart) without correction or with contact lens correction, and stereoscopic acuity at near of 50 arc seconds or better (measured with the Titmus test). Twenty-five participants were excluded due to their visual acuity not meeting the inclusion criteria. Of the 62 eligible participants, data from 59 individuals were analyzed. Two participants were excluded due to a pupil diameter of less than 4 mm (a technical limitation of the PowerRef 3), and one participant was unable to accomplish the task in VR. Based on the results of the eye examination, all participants were divided into two groups: a control group (n = 26) and a group with binocular and accommodative disorders (n = 33).

This study was approved by the Ethics Committee of the University of Latvia and conducted in accordance with the Declaration of Helsinki. Participants provided written informed consent.

### 2.2. Study Design

First, participants underwent a comprehensive eye examination that included objective and subjective refraction, as well as binocular function assessments at both near and far. The latter included the Worth four-dot suppression test, stereovision evaluations using the Ostenberg and Titmus tests, heterophoria measurements with the von Graefe test, and positive and negative fusional reserves measured with a prism bar. NPC and NPA were assessed using the RAF ruler. The evaluation of accommodation included measuring both positive and negative relative accommodation. Vergence facility (with an 8Δ base in/8Δ base out prism flipper) and binocular accommodative facility (with ± 2.00 D or ± 1.50 D lens flipper) were measured at a 40 cm distance over a period of 60 s. The results of the eye examination were assessed in relation to the clinical norms defined by Scheiman and Wick (2014) [21], and participants were divided into two groups. Participants were classified as having non-strabismic binocular and accommodative disorders if at least two visual functions did not meet the clinical norms. Those who met the clinical norms formed the control group.

Before VR gameplay, subjective and objective measurements of vergence and accommodation responses were taken with participants’ habitual vision correction. Subjective measurements included NPC and NPA. The objective binocular measurements of accommodation responses were performed using the PowerRef 3 device (Plusoptix GmbH, Nuremberg, Germany) in a controlled lighting environment to maintain pupil diameter within the range of 4 mm to 8 mm. The device has a recording frequency of 50 Hz, and an eye refraction measurement range of −7.00 D to +5.00 D. To test accommodation responses to near and far stimuli, a Maltese cross (angular size: 1.8°) was presented at two distances: 33 cm for the near stimulus and 6 m for the far stimulus. Participants were instructed to fixate their gaze on the near stimulus for 10 s, followed by gaze fixation on the far stimulus for 10 s. Both measurements were recorded separately.

Following baseline measurements of vergence and accommodation, participants used the Meta Quest 2 VR headset (Reality Labs, Meta Platforms, Menlo Park, CA, USA) to play Beat Saber (developed by Beat Games) for 20 min. Beat Saber is a game where players slice approaching blocks, requiring convergence and divergence eye movements to track the dynamic visual stimuli. The headset has a resolution of 1832 × 1920 pixels per eye, with a 120 Hz refresh rate. The interpupillary distance (IPD) was individually adjusted by choosing one of three available options that was closest to the participants’ IPD: 58 mm, 63 mm, or 68 mm. Participants were instructed to play Beat Saber while staying in the room area marked for this study.

Upon completion of VR gameplay, participants immediately underwent repeated objective binocular measurements of accommodation responses using the PowerRef 3 device under the same conditions as the initial measurements, followed by subjective measurements (NPC and NPA).

### 2.3. Data Processing and Analysis

Changes in subjective measurements were calculated by subtracting the value following VR from the baseline value. As for objective measurements, blinking data were deleted before analysis. Primary data processing was performed using MATLAB R2020a and Microsoft Excel 2405 with the Real Statistics add-in.

To assess changes in ocular accommodation responses following VR gameplay, shifts in refractive state and changes in accommodation lag were calculated. The equation used to calculate the refraction shift was:

*RefN* = *Ref_postVR_* − *Ref_preVR_* (1)

where *RefN* is the refraction shift (D), *Ref_postVR_* is the objective eye refraction after the VR session (D), and *Ref_preVR_* is the objective refraction before the VR session (D), both measured with the PowerRef 3 device looking at the 6 m distance.

Three types of refraction shifts were set: myopic shift (≤−0.05 D), hyperopic shift (≥0.05 D), and no shift (>−0.05 D and <0.05 D). To calculate the accommodation lag, the following equation was used:

*ALag* = *AD* + *AR*, (2)

where *ALag* is the accommodation lag (D), *AD* is the accommodation demand (D) corresponding to the object’s distance (in this study, 3.03 D was required for a 33 cm distance), and *AR* is the objective refraction measured with the PowerRef 3 device when an individual was looking at a 33 cm distance (D). Next, changes in accommodation lag were calculated using the following equation:

∆*ALag* = *ALag_postVR_* − *ALag_preVR_*, (3)

where ∆*ALag* is the change in accommodation lag (D), *ALag_postVR_* is the accommodation lag following VR gameplay (D), and *ALag_preVR_* is the accommodation lag before VR gameplay (D).

Accommodative microfluctuations (AMFs) before and after VR gameplay were calculated as standard deviation [22]:

(4) $$AMF=\sqrt{\frac{1}{n}}\sum_{i=1}^{n}\;{\left(x_{i}-\bar{x}\right)}^{2}$$

where *AMF* is accommodative microfluctuation (D), *n* is the number of refraction values measured during 10 s, *x_i_* is each individual refraction value, and $\bar{x}$ is the mean refraction value.

The statistical analysis was performed in R 4.2.2. The Shapiro–Wilk normality test was used to verify the normal distribution of data. Extreme outliers were identified using the boxplot method and excluded from further analysis. To assess both subjective and objective measurements, we employed a two-way mixed design analysis of variance (ANOVA), including both a repeated-measurements factor (measurement time) and a between-group factor (participants’ group). A 95% confidence level was set for all analyses.

## 3. Results

Results of both subjective and objective measurements are summarized in Table 1 and analyzed in detail in the following text.

**Figure 1:**
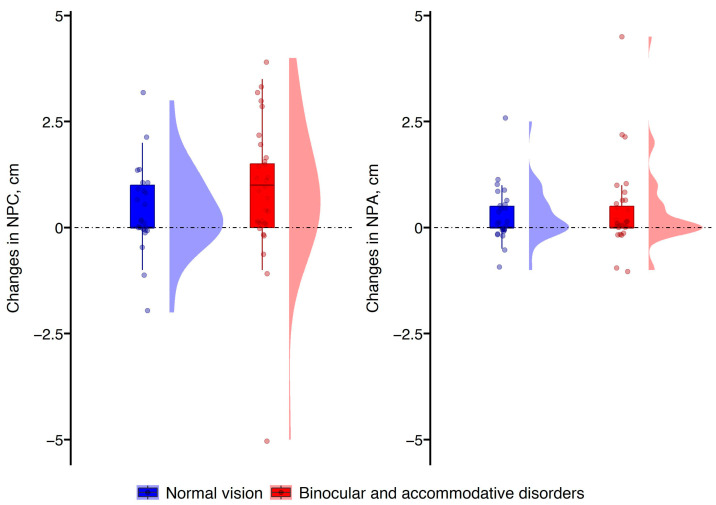
Raincloud plots for changes in subjective measurements following VR gameplay. Positive and negative values, respectively, indicate increased and reduced near point of convergence and near point of accommodation compared to baseline.

Following VR gameplay, subjective measurements showed an increase in values for both the near point of convergence [F(1, 54) = 12.52, *p* = 0.001] and the near point of accommodation [F(1, 55) = 9.56, *p* = 0.003] compared to the baseline. However, the effect of the participants’ group did not reach statistical significance for the near point of convergence [F(1, 54) = 3.72, *p* = 0.059] and the near point of accommodation [F(1, 55) = 2.18, *p* = 0.145]. The distribution of changes in the subjective measurements in both groups of participants can be seen in Figure 1.

Considering individual variations, a reduced near point of convergence following VR gameplay was more common among individuals with binocular and accommodative disorders than in the control group (64% vs. 46%). On average, the near point of convergence changed by 1.8 ± 1.1 cm in the group with binocular and accommodative disorders, and by 1.3 ± 0.7 cm in the control group. However, a reduced near point of accommodation occurred with similar frequency in both groups (36% vs. 42%), with the mean changes being 1.3 ± 1.2 cm in the group with binocular and accommodative disorders and 0.9 ± 0.6 cm in the control group. Overall, larger changes were observed in the near point of convergence compared to those in the near point of accommodation.

**Figure 2:**
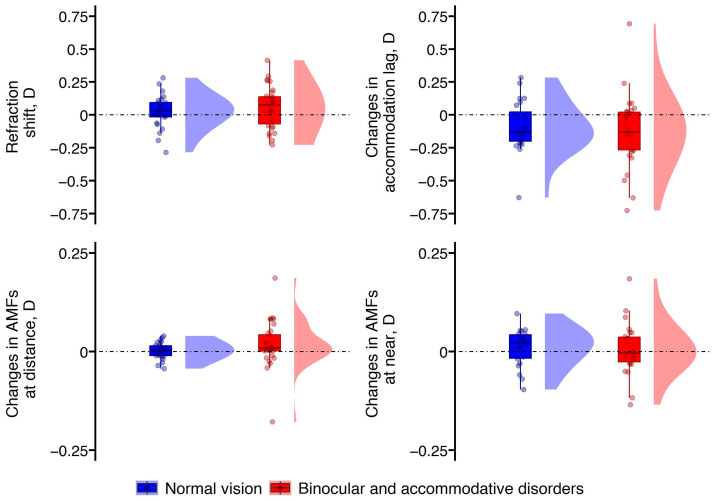
Raincloud plots for changes in objective measurements following VR gameplay.

For objective measurements, we analyzed eye refraction, accommodation lag, and accommodative microfluctuations at both far and near. Significant changes following VR gameplay were revealed in eye refraction [F(1, 54) = 5.13, *p* = 0.028] and accommodation lag [F(1, 51) = 12.43, *p* = 0.001] overall, similarly in both groups of participants, as statistical significance was not reached for the participants’ group factor. Accommodative microfluctuations at near [F(1, 47) = 0.92, *p* = 0.343] did not alter considerably following VR gameplay, and the participants’ group factor did not reach statistical significance for the accommodative microfluctuations at near [F(1, 47) = 0.07, *p* = 0.795]. However, accommodative microfluctuations at far showed a statistically significant change following VR gameplay [F(1, 52) = 8.10, *p* = 0.006]. Additionally, the participants’ group factor also reached statistical significance for accommodative microfluctuations at far [F(1, 52) = 6.34, *p* = 0.015], and significant interaction between the participant group and changes in accommodative microfluctuations at far was observed [F(1, 52) = 4.14, *p* = 0.047]. The distribution of changes in objective measurements in both groups of participants is shown in Figure 2.

Considering individual variations, the majority of participants (69% in the control group and 67% in the group with binocular and accommodative disorders) exhibited a decrease in accommodation lag, indicating a stronger accommodation response to stimuli at a distance of 33 cm. This change was −0.22 ± 0.18 D in the control group and −0.26 ± 0.18 D in the group with binocular and accommodative disorders. Interestingly, a hyperopic shift in refraction was the most prevalent response—approximately half of the participants (46% in the control group and 55% in the group with binocular and accommodative disorders) had it, suggesting a more relaxed accommodation response to distant stimuli compared to the baseline (0.15 ± 0.11 D in the control group and 0.18 ± 0.10 D in the group with binocular and accommodative disorders). Meanwhile, 23% of participants in the control group and 27% of those with binocular and accommodative disorders showed a myopic shift in refraction following VR gameplay (−0.14 ± 0.08 D in the control group and −0.13 ± 0.06 D in the group with binocular and accommodative disorders).

## 4. Discussion

Despite VR technology relying on vergence and accommodation mechanisms, VR user studies typically report results from individuals who have good visual acuity and stereo acuity at near and do not have asthenopic complaints [11]. Thus, individuals with non-strabismic binocular and accommodative disorders could have been included in these studies, as mild disorders might not cause asthenopic complaints or may even be unnoticeable to these individuals. Our study has shown that individuals with non-strabismic binocular and accommodative disorders exhibit larger variations in vergence and accommodation responses as well as less stable refraction responses to distant stimuli following VR gameplay.

The presentation of three-dimensional digital information in VR can challenge the visual system when images appear at various distances from the focal plane. In line with other studies [4,5,6], we observed a reduction in near points of convergence and accommodation following VR gameplay. This reduction in oculomotor function is often attributed to vergence–accommodation conflict, which can occur in VR environments. Our findings complement this by showing that individuals with non-strabismic binocular and accommodative disorders can be more prone to changes.

Most evidence regarding altered vergence and accommodation responses following VR usage is typically based on subjective measurements, leaving it unclear whether objective measurements could provide a more comprehensive assessment of the underlying visual processes. To address this, we used the PowerRef 3, an eccentric photorefractor, to obtain objective measurements of eye refraction while viewing stimuli at various distances, allowing for a more detailed assessment of accommodative behavior. This approach proved effective to some extent, as changes were observed in accommodation lag, refractive state, and accommodative microfluctuations at far.

Interestingly, considerable differences were revealed comparing accommodative microfluctuations at far between the two groups of participants. Accommodative microfluctuations are small, rapid variations in the eye lens’ refractive power typically not exceeding 0.50 D [23]. These play an important role in maintaining clear vision while adjusting to changing visual demands, and reflect the stability of accommodative response [24]. In this study, individuals with non-strabismic binocular and accommodative disorders had larger accommodative microfluctuations at far that were more altered following VR gameplay, indicating difficulties in maintaining the steady-state refractive response.

The results of this study should be interpreted considering its limitations related to the exploratory study design. First, the group of participants with non-strabismic binocular and accommodative disorders was heterogeneous in terms of baseline vergence and accommodation responses. Future research should focus on how users with specific diagnoses experience VR. Second, the type of visual stimuli used in this study presents a limitation. The Beat Saber game, commonly used to explore vergence and accommodation responses to VR, despite being described as an exergame [5] and actively stimulating vergence eye movements, cannot be perceived as a controlled viewing condition, and the vergence–accommodation conflict is not calculated. Third, measurements were conducted before and after VR gameplay due to the closed nature of the headsets. It is known that changes in vergence and accommodation responses are temporary and can return to baseline after VR exposure [5,6]. In the future, with technological advancements, it would be beneficial to measure vergence and accommodation responses during VR gameplay to better understand the adaptation processes of the visual system.

Overall, our study indicated that objective measurements could serve as a valuable complement to subjective measurements, facilitating the detection of changes in vergence and accommodation responses following VR exposure that might otherwise remain undetected. Despite the absence of statistically significant differences in most of the assessed parameters, individuals with non-strabismic and accommodative disorders exhibited greater variability in near point of convergence and accommodation lag. These findings indicate that paying attention to individual differences in vergence and accommodation responses may help predict how these will be altered following VR use.

## Figures and Tables

**Table 1:** Results of subjective and objective measurements for participants in the control group and those with binocular and accommodative disorders presented as the mean (SD).

Parameter	Control Group		Binocular and Accommodative Disorders	
	Before VR	After VR	Before VR	After VR
NPC, cm	6.9 (1.8)	7.3 (2.2)	8.0 (2.9)	8.8 (3.0)
NPA, cm	9.7 (1.3)	10.0 (1.3)	10.3 (2.0)	10.7 (2.2)
Eye refraction, D	−0.23 (0.44)	−0.21 (0.45)	−0.29 (0.38)	−0.24 (0.36)
Accommodation lag, D	1.49 (0.31)	1.40 (0.44)	1.56 (0.37)	1.42 (0.28)
AMFs at far, D	0.06 (0.02)	0.06 (0.02)	0.06 (0.02)	0.08 (0.03)
AMFs at near, D	0.12 (0.05)	0.13 (0.04)	0.12 (0.06)	0.13 (0.04)

Abbreviations: AMFs—accommodative microfluctuations, cm—centimeter, D—diopter, NPC—near point of convergence, NPA—near point of accommodation, VR—virtual reality.

## Data Availability

The original contributions presented in the study are included in this article, further inquiries can be directed to the corresponding author.
